# An *NOS3* Haplotype is Protective against Hypertension in a Caucasian Population

**DOI:** 10.4061/2010/865031

**Published:** 2010-03-25

**Authors:** Georgios D. Kitsios, Elias Zintzaras

**Affiliations:** ^1^Institute for Clinical Research and Health Policy Studies, Tufts Medical Center, Tufts University School of Medicine, 800 Washington Street, Tufts MC no. 63, Boston, MA 02111, USA; ^2^Department of Biomathematics, University of Thessaly School of Medicine, 2 Panepistimiou, Biopolis, Larissa 41100, Greece

## Abstract

The endothelial nitric oxide synthase gene (*NOS3*) has been implicated in the development of hypertension, although the specific role of variants and haplotypes has not been clarified. In this study, the association of three polymorphisms (promoter T786C, intronic 4a/b, and nonsynonymous G894T) was tested in a case-control sample of 230 patients with essential hypertension and 306 healthy controls. Haplotype analysis was also performed. The mutant allele a^∗^ of the 4a/b polymorphism showed a protective effect against hypertension under a dominant model (odds ratio = 0.64, 95% confidence interval (0.44–0.93)), although this effect was not significant after the adjustment for covariates (*P* = 0.06). The estimated frequency of the haplotype composed of the T786^∗^, 4a^∗^, and G894^∗^ alleles was significantly higher in controls (5.5%) compared to cases (2%). These results indicate that although individual *NOS3* polymorphisms are not associated with hypertension, a rare haplotype of the gene might be protective against the development of hypertension.

## 1. Introduction

Hypertension is a multifactorial disorder resulting from complex interactions between genetic and environmental contributions. An increase in vascular resistance and impairment of endothelial-dependent vasodilatation are involved in the pathogenesis of the disease. Nitric oxide (NO) synthesis by the vascular endothelium is important for the regulation of vasodilator tone and control of blood pressure in humans [1, 2]. Given the pivotal role of NO in vascular homeostasis, the endothelial nitric oxide synthase gene (*NOS3*) has emerged as a logical candidate gene in the investigation of hypertension genetics [3]. 

Variants of the *NOS3* gene located in the 7q35-q36 region have been investigated for association with hypertension and other cardiovascular disorders [3]. Among them, three polymorphisms have been widely examined for clinical relevance, based on their potential functional effects and their relatively high minor allele frequency in various ethnic groups [3, 4]: (i) a G894T substitution in exon 7 resulting in a Glu to Asp substitution at codon 298 (rs1799983), (ii) an insertion-deletion in intron 4 (4a/b) consisting of two alleles (the a*-deletion which has four tandem 27-bp repeats and the b*-insertion having five repeats), and (iii) a T786C substitution in the promoter region (rs2070744). In a survey of all published association studies on the relation between the *NOS3* gene polymorphisms and the risk of hypertension, a meta-analysis and subsequent sensitivity analyses supported an association only for the 4a/b polymorphism and hypertension [5]. Most of the studies in the field had investigated the effects of individual polymorphisms and reported marginal or even controversial associations. However, accumulating evidence shows that significant interactions between individual *NOS3 *polymorphisms have a major influence on NO formation [6, 7], and consequently an analysis of haplotypes (and not individual *NOS3* polymorphisms) is expected to be a more powerful approach for detecting genetic susceptibility to hypertension [8, 9]. 

 In order to replicate [10] the association described in the above meta-analysis [5] and explore the effects of *NOS3* haplotypes on the risk of developing hypertension, we performed a case-control study to test for association between *NOS3 *polymorphisms (or haplotypes) and hypertension. We focused on the three previously studied polymorphisms (T786C, 4a/b, and G894T) because of their potential functional implications, their high minor allele frequency, and the extensive publication record on them that would allow for meaningful comparisons [3, 4]. The study presented here was conducted in a case-control sample from a homogeneous population of Caucasian origin (Greeks).

## 2. Materials and Methods

### 2.1. Study Population

All consecutive hypertensive patients presenting in our recruitment centers (primary care practices and outpatient clinics) were systematically evaluated for fulfillment of the following inclusion criteria: (1) patient age more than 20 years, (2) age-at-onset of hypertension before 60 years of age (in order to obtain a phenotype with enriched genetic component) [11], (3) established hypertension defined either as long-term treatment of the disease or in those previously untreated with systolic blood pressure (SBP) more than 140 mmHg or diastolic blood pressure (DBP) more than 90 mmHg, (4) documented absence of secondary forms of hypertension after clinical and laboratory work up (such as renal, renovascular or endocrine disease) and (5) absence of diabetes mellitus. A total of 230 nondiabetic patients with essential hypertension were finally recruited. Each patient's medical history was obtained using a standardized questionnaire regarding the lifestyle, smoking (current or ex-smokers), alcohol or drug intake. Medical history of hypertension, cardiovascular disease, hyperlipidemia, and current medications was recorded. A complete physical examination was performed including measurement of supine SBP and DBP (*using mercury* column sphygmomanometers), electrocardiogram recording, and measurement of somatometric parameters (height, weight, body mass index (BMI)). Spouses and friends of cases were interviewed to assess their appropriateness for inclusion as a healthy control group. A group of 306 normotensive, healthy control subjects was also recruited according to the following criteria: (1) subject age more than 20 years, (2) absence of hypertension or antihypertensive treatment in the medical history, (3) SBP/DBP less than 140/90 mmHg, respectively, (4) absence of chronic illness (renal, cardiovascular, mental, hepatic, endocrine disorders or cancer) or concomitant chronic medication, and (5) unrelated by blood to cases. A blood sample for biochemical measurements and DNA extraction was taken from each participant. All participants provided written informed consent.

### 2.2. Laboratory Assays

Genomic DNA was extracted from whole blood using the QIAamp DNA blood kit (QIAGEN, Valencia, CA, USA) following the manufacturer's instruction. Genotyping of each polymorphism was performed by amplification from 50 to 100 ng of genomic DNA. The primer sequences used and the laboratory conditions for genotyping (polymerase chain reaction, restriction enzymes, agarose electrophoresis) for each *NOS3* polymorphism have been previously described [12, 13]. Genotyping was performed by laboratory personnel blinded to clinical status.

### 2.3. Statistical Analysis

All statistical analyses were performed using the SAS software (v9.1 SAS Institute Inc, NC, USA). The values for clinical parameters are expressed as mean ± standard deviation. The Student *t*-test and the chi-square test were used for comparisons of continuous and categorical variables, respectively. Any result with *P*  <  .05 was considered statistically significant. The chi-square test with one degree of freedom was used in order to test whether the frequency distribution of genotypes in the control group was in Hardy-Weinberg equilibrium (HWE) (*P*  ≥  .05). Differences in genotype distribution and allele frequencies between hypertensive and control subjects were tested using the chi-square test. The association was expressed as an odds ratio (OR) with the corresponding 95% confidence interval (95% CI). Multivariate logistic regression analysis was also used to adjust for the effects of covariates (age, gender, BMI, and smoking status) on the genotype-phenotype association. Addition of a squared term of age was found to improve model fit, as tested with the likelihood ratio test, and was therefore kept in the final model. For the regression models, the genetic effect of the mutant allele (786C*, 4a*, and 894T*, resp.) was assumed to be dominant (mutant carriers versus wild type homozygotes), or recessive (mutant homozygotes versus wild type carriers), or additive (mutant homozygotes versus wild type homozygotes) or codominant (heterozygotes versus homozygotes), for each *NOS3* polymorphism [10]. Linkage disequilibrium (LD) and haplotype analysis were performed using the SHEsis and HAPSTAT software platforms [14, 15]. The order of variants in the inferred haplotypes was T786C, 4a/b, G894T, corresponding to the physical location of these variants in the *NOS3* gene. The threshold haplotype frequency value for inclusion in the analysis was set at 3%. Sample size calculations were performed by using the Power Calculator for Genetic Studies [16]. A modest genetic effect (OR = 1.3), and observed disease prevalence (30%), and minor allele frequencies (786C*  =  40%, 4a*  =  20% and 894T*  =  35%) in Caucasian populations were imputed [17]. Sufficient power (>80%) to detect modest genetic effects was achieved with a sample of 230 cases and 306 controls for all three polymorphisms. A systematic review of the literature for studies investigating the association between *NOS3* haplotypes and hypertension was also conducted and the individual study results were summarized.

## 3. Results

The main clinical and demographic characteristics of the subjects are summarized in Table 1. Age, male gender, BMI, SBP, DBP, creatinine, and urea concentration were significantly higher in cases compared to controls. Among cases, 28% suffered from coronary artery disease and almost 90% were treated by antihypertensive medications or statins. 

 Genotyping was successful in 96%, 99%, and 99% of subjects for the T786C, 4a/b and G894T polymorphisms, respectively. The genotype distributions and allele frequencies are shown in Table 2. The genotype distributions in the control group were in HWE for all polymorphisms (*P* = .39, .63 and  .16 for T786C, 4a/b, and G894T polymorphisms, resp.). The genotype distribution differed significantly between cases and controls only for the intronic 4a/b polymorphism (Table 2). In univariate analysis under a dominant model, carriers of the mutant allele a* were 36% less likely to develop hypertension (OR = 0.64, 95% CI (0.44–0.93)) compared to homozygotes of the wild type allele (b*). However, this protective effect of the a* allele was no longer significant after adjustment for possible confounding variables using multiple regression analysis (adjusted OR = 0.52, 95% CI (0.26–1.04)) (Table 3). No significant interaction between the a* allele carriership and smoking was observed (*P* = .44). The remaining analyses, both univariate and multivariate, did not show any significant association for the T786C and G894T polymorphisms and are shown in Table 3. 

 Pairwise LD among the three polymorphisms was measured by the Lewontin standardized disequilibrium coefficient *D*′ and the squared correlation coefficient *r* ^2^ [18], in both groups separately. All pairwise comparisons showed statistically significant LD (*P* < .05), although strong LD (*D*′  >  0.8) was evident only for the 4a/b and G894T polymorphisms in the control group (Table 4).

 The distribution of the estimated haplotype frequencies for cases and controls is presented in Table 5. Six major haplotypes with frequencies >3% were identified. The global chi-square test for association of haplotypes showed that there was an overall significant difference between cases and controls (*P* = .03). This difference was due to the T-a-G* haplotype, which was more frequent in controls (5.5%) compared to cases (2%) (*P* = .02), conferring a protective effect against the development of hypertension. Exploration of possible interactions of the T-a-G* haplotype with confounding variables (age, sex, BMI, smoking status) did not detect any significant results (*P*-values >.05). 

 The literature search identified nine previously published studies on the association between *NOS3* haplotypes and hypertension [6, 7, 19–25] and their results are provided in Table 6.

## 4. Discussion

### 4.1. Novel Findings

This study evaluated relations between common genetic variants and haplotypes in the *NOS3 *gene with essential hypertension. The single locus analysis among the three most commonly studied polymorphisms revealed an association only for the 4a/b polymorphism. Contrary to what was anticipated, carriership of the mutant allele a* was associated with a 36% reduction in the risk of developing hypertension. A meta-analysis conducted by our group has previously shown a detrimental effect for the a* allele; based on unadjusted estimates, the a* allele was associated with an 22% increased risk for hypertension [5]. Interestingly, this association was confined in Caucasians. In the current study, the unadjusted analysis in a Caucasian population revealed an association in the opposite direction (protective effect of the a* allele). However, this association was not significant when the effects of potential confounders were taken into account with a multivariate logistic regression model. Additionally, previous reports have described that the 4a/b polymorphism effects can be smoking-dependent [26, 27]. No significant genotype-smoking interactions were found in our analysis. The lack of association for the G894T and T786C polymorphisms was in concordance with the meta-analysis results [5]. 

 In the haplotype-based association analysis, the distribution of a relatively infrequent haplotype (T-a-G*) was found to be different between cases and controls, resulting in a protective effect against hypertension. The mutant allele of the 4a/b polymorphism is incorporated in this haplotype, along with the two wild type alleles of G894T and T786C polymorphisms. Although not significant in individual analyses, the point estimates of the genetic effects of the alleles involved in this haplotype were also in the protective direction (Table 3). This study could not provide any further information regarding the underlying culprit functional variation captured by this haplotype; however, the LD analysis showed evidence of strong LD between the 4a/b and the G894T polymorphisms in controls, which could potentially pinpoint a locus of interest.

### 4.2. Haplotype Testing in Hypertension

Testing of haplotypes overcomes some of the problems encountered with using single polymorphisms in genetic association studies, because the interaction of multiple genetic markers within a haplotype could be a key determinant of disease susceptibility rather than the individual polymorphism [28, 29]. The haplotype analysis approach is expected to be more powerful than single-marker analysis, because of the ancestral structure incarcerated in the distribution of haplotypes [9, 10]. Particularly if the markers used define mutations within functional DNA then the haplotypes composed of these markers can have more of a biological role. In accordance with this hypothesis, no significant associations were detected for the single-marker analysis promoter (T786C) and the nonsynonymous (G894T) variants used in our study, despite the fact that functionality analyses have demonstrated a role for both variants [3]. 

 Previous studies have yielded contradictory results regarding the role of the T-a-G* haplotype that was found to be protective in our study, as shown in Table 6. Although no correlation with NO production has been detected [7], the T-a-G* has been reported to confer susceptibility to hypertension [19] or to be protective against gestational hypertension and preeclampsia [30]. Since the haplotypic structure has not been previously examined in the Greek population, it is possible that ethnicity may account for the observed discrepancies. The T-a-G* haplotype had a lower frequency in our study (2% and 5.5% in cases and controls) compared to previous reports (ranging from 6% to 20%) [19, 30].

 For complex disorders as hypertension, the rare haplotypes have been recently shown to play a significant role in influencing disease susceptibility [31, 32]. Recent paradigms have also shown that hunting common variation will not probably suffice to track the “missing heritability” explained by variants detected in the genome-wide association era [33, 34]. Multiple rare pathogenic variants are also likely to be important determinants of complex disorders. Although such variants will not be detectable by current techniques based on the use of linked polymorphic markers, advances in genotyping technologies and novel genetic variation maps that capture rare variants (1,000 Genomes Project) will make whole-genome searches for rare variants feasible [35]. Findings from genome-wide agnostic approaches can nevertheless be complemented by prioritized results from additional venues of genomic research (genomic convergence) [36, 37] in order to select for replication studies the candidate genes with the stronger evidence support.

### 4.3. Study Limitations

Large sample sizes and independent replication are sine qua non principles for genetic epidemiology [10, 38]. Our findings are not supportive of a major contributory role of individual genetic polymorphisms but provide evidence for association for a rare haplotype. Given the logistic limitations of single centers, like ours, to recruit large numbers of participants and to replicate the association in multiple cohorts, it is important that the validity of the proposed association here is tested in other studies before scrutinizing the gene in search for causal variants. Additionally, complex disorders such as hypertension are considered to emerge from multiple epistatic and gene-environmental interactions [34]. Although the *NOS3* gene is though to be involved in critical pathway interplays [39], our sample size did not allow the testing for interactions with sufficient power. Finally, the case-control design may have allowed for some elusive misclassification of controls, since the development of the disease is age-related. Replicating our hypothesis in large prospective studies could overcome these unavoidable limitations [38]. 

In conclusion, our genetic association study detected a protective effect of a rare *NOS3* haplotype against hypertension. Although the underlying functional genetic variation in the *NOS3* gene remains to be defined, haplotype-based analyses are expected to be more informative regarding the role of the *NOS3* gene compared to single-marker analyses. Rare haplotypes and variants may not serve as markers of disease with clinical utility on a population-wide basis but they hold the potential to uncover critical pathways involved in the pathogenesis of hypertension and assist in defining novel molecular targets for intervention. Our results require replication in independent cohorts and additional studies in order to disentangle the molecular basis of the detected genetic effects.

## Figures and Tables

**Table 1:** Clinical characteristics of cases and controls.

	Cases (*n* = 230)	Controls (*n* = 306)	*P*-value
Age (years)	63.9 (9.4)	40.8 (18.3)	<.05
Males, *n* (%)	125 (54.4)	68 (22.2)	<.05
BMI	28.7 (4.1)	27.5 (4.4)	<.05
Smokers, *n* (%)	84 (36.5)	80 (29.9)	.08
SBP (mmHg)	145.3 (15.7)	124.4 (12.3)	<.05
DBP (mmHg)	85.1 (10.2)	68.8 (9.7)	<.05
PP (mmHg)	59.3 (17.2)	57.1 (14.1)	.19
CAD, *n* (%)	65 (28.3)	—	N/A
Creatinine (mg/dL)	1.09 (0.8)	0.92 (0.9)	<.05
Urea (mg/dL)	40.8 (8.6)	36.5 (6.6)	<.05
Potassium (mmol/L)	4.3 (0.5)	4.4 (0.3)	.26
Sodium (mmol/L)	138.4 (3.1)	140.0 (4.5)	.38
Total Cholesterol (mg/dL)	190.88 (48.4)	—	N/A
LDL Cholesterol (mg/dL)	113.7 (39.5)	—	N/A
HDL Cholesterol (mg/dL)	46.9 (17.8)	—	N/A
Triglycerides (mg/dL)	132.4 (64.5)	—	N/A
Drug treatment (%)	89.6	—	N/A
ACE-inhibitors (%)	45.2	—	N/A
ARBs (%)	29.0	—	N/A
Beta-blockers (%)	50.3	—	N/A
CCBs (%)	36.8	—	N/A
Diuretics (%)	50.9	—	N/A
Nitrates (%)	13.5	—	N/A
Statins (%)	39.7	—	N/A

Values are mean (standard deviation), unless otherwise specified. Abbreviations: BMI = body mass index, SBP = systolic blood pressure, DBP = diastolic blood pressure, PP = pulse pressure, CAD = coronary artery disease, LDL = low-density lipoprotein, HDL = high density lipoprotein, ACE = angiotensin converting enzyme, ARBs = angiotensin receptor blockers, CCBs = calcium channel blockers, N/A = nonapplicable.

**Table 2:** Genotypic and allelic distributions of *NOS3* polymorphisms for cases and controls.

	Cases, *n* (%)	Controls, *n* (%)	*P*-value
T786C			
TT	69 (30.4)	101 (35.0)	
TC	118 (52.0)	148 (51.2)	
CC	40 (17.6)	40 (13.8)	.37^(a)^
T alleles	246 (55.4)	350 (60.6)	
C alleles	198 (44.6)	228 (39.4)	.12^(b)^

Intron 4a/b			
4b/b	165 (72.4)	190 (62.7)	
4a/b	59 (25.9)	101 (33.3)	
4a/a	4 (1.7)	12 (4.0)	.04^(a)^
b alleles	389 (85.3)	481 (79.4)	
a alleles	67 (14.7)	125 (20.6)	.03^(b)^

G894T			
GG	99 (43.4)	135 (44.7)	
GT	95 (41.7)	130 (43.1)	
TT	34 (14.9)	37 (12.2)	.67^(a)^
G alleles	283 (63.5)	400 (66.2)	
T alleles	163 (36.5)	204 (33.8)	.39^(b)^

^(a)^ *P*-value for the comparison of genotypic distribution. ^(b)^ *P*-value for the comparison of the allelic distribution.

**Table 3:** Unadjusted and adjusted odds ratios with the corresponding 95% confidence intervals for the association of *NOS3* genotypes and hypertension: comparisons for the dominant, recessive, additive, and codominant models of the mutant alleles (786C*, 4a*, 894T*).

Polymorphism	Unadjusted	*P*-value	Adjusted	*P*-value
	OR (95% CI)		OR (95% CI)	
T786C				
Dominant model	1.23 (0.85–1.79)	.28	1.30 (0.64–2.63)	.47
Recessive model	1.33 (0.83–2.15)	.24	0.77 (0.33–1.83)	.56
Additive model	1.46 (0.86–2.49)	.16	0.99 (0.38–2.62)	.98
Codominant model	1.03 (0.73–1.42)	.86	1.48 (0.75–2.93)	.26

Intron 4a/b				
Dominant model	0.64 (0.44–0.93)	.02	0.52 (0.26–1.04)	.06
Recessive model	0.43 (0.14–1.36)	.15	0.49 (0.08–3.07)	.45
Additive model	0.38 (0.12–1.21)	.10	0.40 (0.06–2.78)	.35
Codominant model	0.69 (0.48–1.02)	.06	0.56 (0.28–1.13)	.10

G894T				
Dominant model	1.05 (0.75–1.49)	.77	1.51 (0.78–2.92)	.22
Recessive model	1.25 (0.76–2.07)	.37	1.04 (0.38–2.77)	.94
Additive model	1.25 (0.74–2.14)	.41	1.28 (0.46–3.56)	.64
Codominant model	0.94 (0.67–1.34)	.75	1.51 (0.77–2.96)	.23

**Table 4:** Pairwise linkage disequilibrium metrics [*D*′, (*r* ^2^)] for *NOS3* polymorphisms in cases and controls.

Polymorphisms	Intron 4a/b	G894T
T786C		
Cases	0.46^(a)^ (0.05)^(a)^	0.34^(a)^ (0.09)^(a)^
Controls	0.44^(a)^ (0.15)^(a)^	0.51^(a)^ (0.11)^(a)^

Intron 4a/b		
Cases	—	0.44^(a)^ (0.02)^(a)^
Controls	—	0.82^(a)^ (0.09)^(a)^

^(a)^ *P* < .05.

**Table 5:** *NOS3* haplotype distribution in cases and controls.

Haplotype^(a)^	Cases (%)	Controls (%)	*P*-value
T-b-G*	47.2	43.1	.26
C-b-T*	26.4	20.9	.07
C-a-G*	11.9	13.7	.43
T-b-T*	7.9	11.5	.09
C-b-G*	3.7	4.1	.77
T-a-G*	2	5.5	.02
other	0.9	1.2	—
Global test of association			.03

^(a)^The order of variants in the inferred haplotypes is [T786C-4a/b-G894T] in order to correspond to the physical location of these variants in the *NOS3* gene.

**Table 6:** Summary description of previous studies on the association between *NOS3* haplotypes and essential hypertension.

Study first author, year of publication	Study population ethnicity, (Number of cases/Number of controls)	Evidence for association with *NOS3* haplotypes	Haplotypes with statistically significant results	Direction of haplotypic genetic effect
Sandrim, 2006	Caucasians	Yes	T-b-T*	Protective
[21]	(112/113)		C-b-G*	Protective
			C-b-T*	Susceptibility
	Blacks (91/87)	Yes	T-b-T*	Protective
			C-b-G*	Protective

Sandrim, 2006	Mixed	Yes	C-b-G*	Protective
[22]	(119/102)		C-b-T*	Susceptibility

Zhao, 2006	East-Asians	No		
[23]	(503/490)			

Sandrim, 2006	Mixed	Yes	C-b-G*	Protective
[24]	(216/111)		C-b-T*	Susceptibility

Sandrim, 2007	Mixed	Yes	C-b-G*	Protective
[7]	(154/98)		C-b-T*	Susceptibility

Nejatizadeh, 2008 [6]	Indian (455/345)	Yes	T-a-G*	Protective
			T-a-T*	Protective
			C-a-G*	Protective
			T-b-G*	Susceptibility

Conen, 2008 [25]	Caucasian (18436)	No		

Kumar, 2009 [19]	Indian (440/470)	Yes	T-a-G*	Susceptibility
			T-a-T*	Susceptibility
			C-a-G*	Susceptibility
			T-b-G*	Protective

Vasconcellos, 2010 [20]	Caucasians	Yes	C-b-G*	Protective
	(173/101)		C-a-G*	Susceptibility
